# Prevention of Peritoneal Metastases from Colon Cancer in High-Risk Patients: Preliminary Results of Surgery plus Prophylactic HIPEC

**DOI:** 10.1155/2012/141585

**Published:** 2012-05-08

**Authors:** Paolo Sammartino, Simone Sibio, Daniele Biacchi, Maurizio Cardi, Fabio Accarpio, Pietro Mingazzini, Maria Sofia Rosati, Tommaso Cornali, Angelo Di Giorgio

**Affiliations:** ^1^Dipartimento di Chirurgia Pietro Valdoni, Azienda Policlinico Umberto I, Università degli Studi di Roma “La Sapienza,” 00161 Roma, Italy; ^2^Dipartimento di Medicina Sperimentale, Azienda Policlinico Umberto I, Università degli Studi di Roma “La Sapienza,” 00161 Roma, Italy

## Abstract

The study compared the outcome in patients with advanced colonic cancer at high risk of peritoneal metastases (mucinous or signet-ring cell) without peritoneal or systemic spread, treated with standard colectomy or a more aggressive combined surgical approach. The study included patients with colonic cancer with clinical T3/T4, any N, M0, and mucinous or signet ring cell histology. The 25 patients in the experimental group underwent hemicolectomy, omentectomy, bilateral adnexectomy, hepatic round ligament resection, and appendectomy, followed by HIPEC. The control group comprised 50 patients treated with standard surgical resection during the same period in the same hospital by different surgical teams. Outcome data, morbidity, peritoneal recurrence rate, and overall, and disease-free survival, were compared. Peritoneal recurrence developed in 4% of patients in the experimental group and 22% of controls without increasing morbidity (*P* < 0.05). Actuarial overall survival curves disclosed no significant differences, whereas actuarial disease-free survival curves showed a significant difference between groups (36.8 versus 21.9 months, *P* < 0.01). A more aggressive preventive surgical approach combined with HIPEC reduces the incidence of peritoneal recurrence in patients with advanced mucinous colonic cancer and also significantly increases disease-free survival compared with a homogeneous control group treated with a standard surgical approach without increasing morbidity.

## 1. Introduction

Epidemiological data indicate that peritoneal spread from colorectal cancer is an event that involves 10–15% of patients at the time of primary cancer resection and about 25–50% of patients with recurrent disease, generally leading to death within weeks or months [1–5]. Several features of primary tumors of colorectal origin appear to be related to a later development of peritoneal spread: mucinous colorectal cancers or signet ring cell carcinomas tend preferentially to metastasize to the peritoneum or ovaries [6–10]. Intraperitoneal metastases may spread by full-thickness bowel wall invasion or may arise iatrogenically during surgery when “in transit” tumor cells or emboli escape from dissected lymph vessels within the bowel lumen or reach the peritoneal cavity through blood spill from the surgical field [11].

Since the 1990s, Paul Sugarbaker's studies on cytoreductive surgery plus perioperative intraperitoneal chemotherapy such as hyperthermic intraperitoneal chemotherapy (HIPEC) have prompted a new treatment option for selected patients with peritoneal metastases from colorectal cancer [12–14]. Three studies, one randomized and two nonrandomized, have shown that this combined procedure provides a better outcome than 5-fluorouracil-based chemotherapy or more modern chemotherapy regimens [15–17].

Despite these encouraging results, even if 5-year survival can reach a value close to 45% at the expense of a mortality rate ranging from 3 to 5% and a morbidity rate around 30% in selected cases [18, 19], outcome depends on many factors. The first is peritoneal involvement as measured by the peritoneal cancer index (PCI), the second the degree of cytoreduction achieved, and finally the surgical team's level of experience [20, 21]. Given that better prognostic results can be expected only in patients with a low PCI, in whom complete cytoreduction is possible, and because current imaging techniques cannot detect peritoneal metastases before they becomes clinically evident and symptomatic [22, 23], in practice these combined approaches apply only to few patients and rarely offer long-term survival.

In the past, in line with what others now propose for ovarian cancer [24], experimental investigations and clinical trials have used normothermic intraperitoneal chemotherapy as adjuvant treatment in colorectal patients at high risk of recurrence, with inconclusive results [25–27]. In recent years, some have suggested early second-look surgery in the absence of clinical signs of recurrence for colorectal patients at high risk of peritoneal relapse to detect and treat those with carcinomatosis at an initial stage [28, 29]. Based on preliminary results, two randomized trials will begin in France and the United States to answer the question whether second-look surgery envisaging cytoreductive surgery with HIPEC or HIPEC alone will prolong overall survival and reduce the risk of relapse compared with standard care (observation) in patients with colorectal cancer at high risk for peritoneal spread [30, 31].

Prompted by the need to seek new ways of managing colorectal cancer in patients at high risk of recurrence but still without evident signs of peritoneal spread, we decided to concentrate our efforts on a timely strategy envisaging a primary operation aimed at preventing peritoneal metastases. Ample evidence shows that the two major elements influencing peritoneal spread in colorectal cancer are the depth of bowel wall invasion (pT3/4) and histological features of the malignancy (mucinous and signet ring cell carcinomas) [5–11]. Both are characteristics that the surgeon can verify during the primary operation and if necessary use the information to change the strategy to a more aggressive approach combining surgical resection and HIPEC.

We designed this single-center case-control study to analyze outcome in two comparable groups of patients with advanced colonic cancer (pT3/4 with mucinous or signet ring cell cancer) without peritoneal or systemic spread treated with standard colectomy, according to the established guidelines [32] (control group), or by a more aggressive combined approach aimed to prevent peritoneal spread. For this purpose, in the experimental group we extended our standard surgical resection to the metastatic sanctuaries of peritoneal diffusion (including the omentum, adnexa, and appendix) and combined these surgical procedures with prophylactic HIPEC. As the primary outcome variables we compared morbidity, the incidence of peritoneal recurrence and overall and disease-free survival in the two groups.

## 2. Materials and Methods

### 2.1. Experimental Group

 The study included patients with colonic cancer or intraperitoneal rectosigmoid cancer (over 15 cm from the anal verge) with clinical T3/T4, any N, and M0 stage treated at the Department of Surgery Pietro Valdoni at Sapienza University of Rome from January 2006 to December 2008. To avoid bias from neoadjuvant therapy we excluded patients with extraperitoneal rectal cancer. Selection criteria were age younger than 70 years, cancer with mucinous or signet ring cell components (>20% according to criteria proposed by Ogino et al. [33], performance status 0–2 (WHO) [34]), and adequate renal, hepatic, and bone marrow function. All patients gave specific informed written consent. Exclusion criteria were metastatic disease, other malignances, multiple colorectal cancer, active infections, or severe associated medical conditions. Patients with perforated cancers were considered eligible regardless of histology. At surgical exploration, patients with unrecognized peritoneal seedings were excluded from the study as well as those with hepatic involvement detected at intraoperative ultrasound. None of the patients underwent peritoneal lavage cytology [35]. At operation, after the standard hemicolectomy done according to the established guidelines, intraoperative pathologic evaluation assessed tumor depth (pT) and the histologic features necessary to include the patient in the study. In the selected cases the surgical resection also included complete omentectomy, bilateral adnexectomy in postmenopausal patients, hepatic round ligament resection and appendectomy if not already done. At the end of surgery, HIPEC was delivered with the closed technique with oxaliplatin 460 mg/m^2^ in 2 l/m^2^ of dextrose at a temperature of 43°C over 30 minutes at a flow rate of 2 L/min. Before HIPEC began and during surgery patients received intravenous fluorouracil of 400 mg/m^2^ and leucovorin of 20 mg/m^2^ to potentiate oxaliplatin activity. Systemic adjuvant chemotherapy was reserved after discharge to patients with pT4, node positive, and G3 tumors. The study was approved by the hospital institutional review board.

### 2.2. Control Group

 Control subjects were retrospectively selected from patients with colonic cancer treated with standard surgical resection, during the same period in the same hospital but by different surgical teams. The selection process comprised two steps. During the first step surgeons from another surgical team in our hospital selected from their records all patients with colonic cancer treated from January 2006 to December 2008 and who met the eligibility criteria required in our experimental study and had known follow-up. In particular, we selected patients with T3/T4 mucinous or signet ring cell carcinoma resected for cure (R0) without systemic spread. As in the experimental group, control patients with perforated colon cancer were included regardless of histology. During the second step the principal investigator (P. Sammartino) double-checked the medical records for the potentially eligible patients provided by other surgical teams by recontacting the investigator to ensure that the eligibility criteria had been homogeneously applied. During double-checking the investigator was unaware of the patients' outcome.

### 2.3. Follow-Up and Statistics

Data for patients in the experimental group were recorded prospectively in a specific database. Data for patients the control group were recorded retrospectively. Surgical complications and adverse events were monitored in both groups and graded from 0 to V in accordance with the National Cancer Institute Common Toxicity Criteria [36]. Follow-up assessments took place every 3 months with clinical evaluation and tumor marker monitoring. A 64-section multidetector computed tomography (MDCT) and magnetic resonance imaging (MRI) with conventional and diffusion-weighted sequences were obtained alternatively every 6 months in the experimental and control groups, according to a protocol developed in collaboration with a dedicated radiological team [37]. The definition of peritoneal recurrence, included imaging findings of locoregional progression as well as peritoneal metastases distant from the resection site. No patient was excluded from the survival analysis. The chi-square test was used for univariate comparison. Survival curves were calculated with the Kaplan-Meier method and compared with the log-rank test. Survival was measured from the date of surgical treatment until death or the last follow-up. *P* values < 0.05 were considered to indicate statistical significance.

## 3. Results

Of the 230 patients with colonic cancer treated in our department between January 2006 and December 2008, 25 fulfilled the inclusion criteria and agreed to take part in this experimental investigation. A total of 75 patients were proposed for matching with those in the experimental study and after double checking for eligible criteria 50 were included in the control group. The clinical characteristics for both groups are shown in Table 1. Surgical procedures performed in both groups are reported in Table 2.

 When surgery ended all 25 patients in the experimental group underwent HIPEC. In the study group a mean of 20 lymph nodes per patient were removed (range 15–28) and in the control group a mean of 19 (range 14–31). Locoregional lymph node metastases were found in 34% of patients in the experimental group and in 28% of those in the control group. Anatomopathological studies in the experimental group showed that none of the surgical specimens excised according to the protocol contained malignant disease. All the surgical procedures in both groups were R0. The mean length of surgery, blood loss, and postoperative stay were similar in the two groups. Except for 1 patient in the experimental group who had grade 2 pancreatitis related to HIPEC toxicity (that promptly regressed after medical therapy) morbidity rates were similar in the two groups. One patient in the experimental group underwent emergency laparotomy on postoperative day 2 for bleeding. One patient in the control group had a grade III complication (left ureteral leakage) that required endoscopy to place a stent, and 3 patients underwent a second laparotomy to construct an ileostomy for anastomotic leakage (Table 3). A total of 13 patients in the experimental group (52%) and 23 in the control group (46%) underwent first-line systemic adjuvant chemotherapy with fluorouracil and oxaliplatin. In relapsed patients second-line chemotherapy included irinotecan or molecular target drugs (cetuximab and bevacizumab or both).

### 3.1. Follow-Up

 After a mean 37.8-month follow-up in the experimental group and 35.1 months in the control group, 24% in the experimental group and 32% of the controls had recurrent disease (Table 4).

### 3.2. Experimental Group

 Six patients showed relapse of disease: 5 had hepatic and or pulmonary metastases (mean time of recurrence 13 months) and 1 developed a peritoneal recurrence. This patient underwent a right hemicolectomy with abdominal wall resection for a T4b tumour and experienced a peritoneal recurrence detected at 30 months after operation. Two patients (one with single hepatic metastases and the peritoneal recurrence) underwent a second surgical procedure and are at the moment alive and disease-free at 38 and 39 months. Of the other 4 patients, 1 is currently alive with hepatic and pulmonary metastases and 3 died from progressive disease at a mean of 21 months.

### 3.3. Control Group

 Sixteen patients showed recurrent disease: a peritoneal recurrence was found in 11 (22%), associated in 4 cases with hepatic and/or pulmonary disease at a mean of 12.7 months after first operation. Initial diagnosis of peritoneal metastases was made after MDCT and MRI findings according to our published protocol [37]. In 9 patients the diagnosis was also confirmed by endoperitoneal ascites cytology or histology during laparoscopy or operation. Five patients showed only a systemic progression of the disease (hepatic in 3, pulmonary in 1, and both in 1). Three patients underwent a second surgical procedure: 1 underwent a wedge resection for a single hepatic metastasis (alive disease-free at 31 months) and 2 cytoreduction and HIPEC for peritoneal metastases (alive with disease at 26 and 24 months). Four patients are currently alive with disease and 9 died from progression at a mean of 15.6 months.

**Figure 1:**
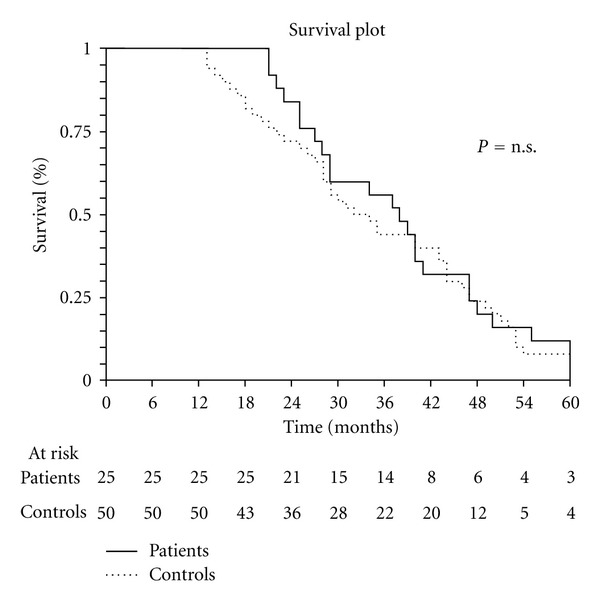
Overall survival.

**Figure 2:**
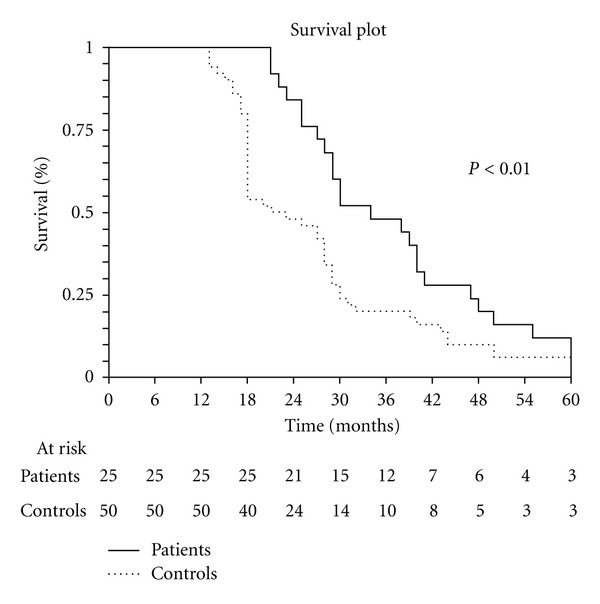
Disease free survival.

 A statistically significant difference in development of peritoneal metastases was observed between the 2 groups (*P* < 0.05, Table 4). The actuarial overall survival curves disclose no significant difference between the two groups (Figure 1) whereas the actuarial disease-free survival curves already show a significant difference (*P* < 0.01) between the two groups (36.8 months in the experimental group versus 21.9 months in the control group, Figure 2).

## 4. Discussion

Our preliminary results in this single-center case-control study show that our more aggressive preventive surgical approach combined with HIPEC significantly reduces the incidence of peritoneal recurrence in patients with advanced mucinous colonic cancer and also significantly increases disease-free survival compared with a homogeneous control group treated with a standard surgical approach and does so without increasing morbidity. Although our current data for overall survival seem as yet to show no difference between the survival curves for the experimental group and controls, the significant difference in disease-free survival suggests that overall survival will eventually differ as follow-up progresses.

 Our preventive surgical strategy could have improved outcome because it is based on current knowledge on peritoneal fluid dynamics showing that exfoliated tumor cells from full-thickness tumors especially those from mucinous histotypes colonize specific sites [38, 39]. According to a series of well-documented events, as in ovarian cancer progression, malignant spread in colonic cancer preferentially targets the omentum, pelvis, and ileocecal regions. Removing these target organs at the first surgical intervention regardless of whether they are macroscopically involved and following these surgical procedures with HIPEC, both aimed to eradicate microscopic residual disease, seems a reasonable strategy to reduce peritoneal spread.

 We cannot say whether the reduced peritoneal recurrence and significantly improved disease-free survival in the experimental group depend on the associated surgical procedures (omentectomy, adnexectomy, appendectomy, and round ligament resection) or on HIPEC. Although pathological studies of the anatomic structures preventively removed in our patients disclosed no evidence of malignant disease, we can reasonably presume that removing these structures and delivering HIPEC both contributed to preventing microscopic peritoneal diffusion [40, 41].

 Our proposal to address the problem from a new angle, namely, preventing colorectal peritoneal spread, seems to offer a promising alternative to those who recommend an early second look in high-risk patients [28, 31]. In our preventive study we defined high-risk patients with advanced colonic cancer as those with pT3/4 mucinous or signet ring cell cancer without peritoneal or systemic spread whereas those proposing second-look surgery enrolled a varied population including patients who at the primary intervention already had limited peritoneal carcinomatosis or ovarian metastases [28, 31]. These nonhomogeneous populations will make it difficult to interpret outcomes in the two ongoing randomized trials investigating second-look surgery [30, 31]. A major concern is whether randomizing patients to second-look surgery or observation is ethically justifiable given that in a preliminary report Elias et al. at second-look found that more than 50% of high-risk patients had peritoneal carcinomatosis that clinical and imaging examination left unrecognized [28]. Lastly, another problem related to second-look surgery is that a whole class of patients (those termed at high risk) must be referred to highly specialized tertiary centers (peritoneal surface malignancy treatment centers) so that peritoneal carcinomatosis if found can be properly treated. As cancer surgeons well know, the medical community still regards integrated treatments for peritoneal carcinomatosis with skepticism. And we all know how difficult it is to persuade patients (and their oncologist) to undergo a second intervention that may be lengthy and not without risks in the absence of specific symptoms and documentable clinical evidence. From the viewpoint of feasibility and costs we therefore consider it more appropriate to concentrate our efforts on and invest our resources in preventing peritoneal carcinomatosis right from the primary operation. If our innovative preventive strategy proves therapeutically worthwhile then it could be done in a larger number of surgical centers, would involve a larger number of patients, and might finally change the therapeutic options available to patients with advanced colorectal cancer at risk for peritoneal carcinomatosis [29]. 

Some might criticize our preventive proposal stating that in patients with advanced colonic cancer with no documented signs of carcinomatosis our aggressive approach could be considered overtreatment. This criticism notwithstanding, our early aggressive approach receives strong support because without increasing morbidity rates it lowers the incidence of peritoneal carcinomatosis and offers better disease-free survival than in a homogeneous sample of patients who received standard surgical treatment. Our preventive approach also accords with Sugarbaker, who recommended after second-look surgery negative for carcinomatosis a procedure analogous to the one we describe here (omentectomy, adnexectomy, and HIPEC) [29]. Hence, in high-risk patients why not use this approach right from the primary surgical intervention. The true therapeutic value of our preventive surgical approach for patients with advanced mucinous colonic cancer awaits confirmation in future randomized multicenter studies.

## Figures and Tables

**Table 1:** Clinical characteristics of the 2 groups.

	Patients (25)		Controls (50)	
	Mean age 62 (45–70)		Mean age 63 (48–72)	
	*N*	%	*N*	%
Sex				
Male	16	66	31	62
Female	9	34	19	38
Performance status				
0	21	84	41	82
1	4	16	7	14
2	—	—	2	4
Tumor site				
Right colon	9	36	15	30
Transverse colon	3	12	6	12
Left colon	13	52	29	58
Pt				
pT3	19	76	40	80
pT4a	1	4	1	2
pT4b	5	20	9	18
Nodal status				
N0	16	66	36	72
N1-2	9	34	14	28
Grading				
G2	17	68	37	64
G3	8	32	13	26
Histology				
Mucinous	23	92	45	90
Signet ring cell	1	4	4	8
Adc nos	1*	4	1*	2

*Perforated patients.

**Table 2:** Surgical procedures performed in the 2 groups.

	Patients	Controls
Surgical procedures		
Complete omentectomy	25*	—
Hepatic round ligament resection	25*	—
Left hemicolectomy	13	29
Appendectomy	10*	—
Right hemicolectomy	9	15
Bilateral adnexectomy	6*	1^°§^
Transverse colon resection	3	6
Cholecystectomy	2^§^	2^§^
Abdominal wall resection	2°	3°
Small bowel resection	2°	3°
Right adnexectomy	1*	1°
Hysterectomy	1^§^	1°
Total, mean per patient	99–3.9	61–1.2

*Procedures performed according to the study protocol.

°Procedures performed for the tumor direct invasion.

^§^Procedures performed for the coexisting benign disease.

**Table 3:** Surgical outcome in the 2 groups.

Morbidity	Patients		Controls	
	*N*	%	*N*	%
Grade I/II	3	12	5	10
Grade III	—	—	1	2
Grade IV	1	4	3	6
Hipec tox. grade 2	1*	4	—	—

	Patients		Controls	

Mean operation time (min)	180 (120–210)		155 (90–220)	
Mean blood loss (mL)	210 (180–290)		230 (150–400)	
Postop. stay (days)	11 (8–14)		14 (8–21)	

*Pancreatic.

**Table 4:** Site of recurrence.

	Patients (25)		Controls (50)		*P*
Metastases	*N*	%	*N*	%	
Distant	5	20	9	18	ns
Peritoneal	1	4	11*	22	<0.05
Total	6	24	16	32	ns

*4 patients had also distant metastases.
